# Supplementary material for: Are carbon emissions trading and green financial instruments synergistic? -Comprehensive quantitative research based on content analysis
Source: PLoS One. 2024 Mar 7;19(3):e0298601. doi: 10.1371/journal.pone.0298601 (PMC10919601; doi:10.1371/journal.pone.0298601)
Supplement: S1 Data — (ZIP) [file pone.0298601.s001.zip › Data/地级市Z_F工作报告.zip!/╕╩╦α/╢¿╬≈╩╨/└┤╫╘╣1⁄2╓┌║┼í╛┬φ┐╦╩2╛▌═°í┐_2014.docx]

各位代表：

　　现在，我代表市人民政府，向大会作工作报告，请予审议，并请各位政协委员和列席会议人员提出意见。

　　2013年工作回顾

　　过去的一年，在省委、省政府和市委的坚强领导下,全市上下认真贯彻党的十八大、十八届三中全会和省第十二次党代会、市第三次党代会精神，努力克服地震、冰雹、暴洪、干旱等自然灾害带来的严重影响，统筹推进扶贫攻坚与小康建设、救灾重建与经济增长、社会建设与民生改善，经济社会保持平稳健康发展。预计全市完成生产总值250亿元，增长11%左右。固定资产投资509亿元，增长30%。社会消费品零售总额82亿元，增长15%。大口径财政收入30.7亿元，公共财政预算收入17亿元，公共财政预算支出176.9亿元，分别增长32.1%、30%和19%。城镇居民人均可支配收入16137元，增长13%。农民人均纯收入4190元，增长16%。金融机构存贷款余额分别为518亿元和315亿元，分别增长26.4%和31.6%。居民消费价格涨幅3.4%，人口自然增长率6‰，城镇登记失业率4%，节能减排任务全面完成。

　　一年来，我们主要做了以下工作：

　　（一）科学组织救灾重建。去年的定西灾害频发。7月22日，岷县漳县6.6级地震顷刻成灾；8月23日，安定特大暴雨突如其来；9月16日，暴洪泥石流再袭地震灾区。纷至叠加的灾害，给全市人民群众生命财产造成重大损失，105个同胞鲜活的生命瞬间被灾难吞噬。灾害发生后，全市上下快速反应、紧急动员、科学应对，全力将灾害损失减少到最低程度。特别是岷县漳县6.6级地震发生后，我们认真贯彻习近平总书记等中央领导的重要批示和李克强总理视察灾区时的重要讲话精神，按照省委、省政府和市委的部署要求，始终把抢救生命放在第一位，迅速启动应急预案，紧急运送救治伤员，全力搜救失踪人员，抓紧抢修基础设施，全方位开展卫生防疫，严密防范次生灾害，灾后32小时内失踪、被埋人员全部找到，22小时内损毁道路全部打通，34小时内受损电力、通讯设施恢复运营，伤员救治零死亡。建立和落实市县领导包抓责任制，设立救灾物资直供点，强化价格监管，调拨搭建帐篷3.5万顶，发放救助金3.5亿元，131所严重受损学校如期开学，灾区生产生活秩序在最短时间内得以恢复，26.9万受灾群众温暖安全过冬得到有效保障。灾后恢复重建顺利启动，重建实施方案和重灾村、集中安置点、小城镇建设等重建规划编制加快推进，居民住房重建开工率33%。“5.10”岷县特大冰雹山洪泥石流灾后恢复重建的318个项目已开工309个，完工236个，到位资金24.2亿元，累计完成投资19.4亿元，占规划总投资的65%。

　　（二）强力推进项目建设。落实项目前期经费6560万元，谋划生成“3341”项目工程入库项目4642个，总投资7272亿元。争取国家政策性资金86.9亿元，增长32.2%。《甘肃定西渭河源区生态保护与综合治理规划》已经国家发展改革委批复，《定西市生态文明建设规划》通过国家发展改革委组织的专家审查。实施500万元以上项目1666个，较上年增加340个，其中亿元以上250个，较上年增加95个，完成投资248亿元。临渭高速、马云二级公路开工建设，文殪、定临二级公路及安定过境段建成通车，兰渝铁路、宝兰客专、兰州至定西输气管道等省属重大项目进展顺利。中盛铝业25万吨铝型材等100个市列重点推进项目和陇西中天红芪口服液等100个市上督导、县区推进的重点项目进展良好。加强和改进招商引资工作，引进投资项目337个，到位资金298亿元，增长63%。积极参加第19届兰洽会暨首届民企陇上行活动，签约项目131个，总投资581亿元，到位资金138亿元，创历年新高。我市重点项目建设工作得到全省项目建设观摩活动与会代表及省委、省政府高度评价。

　　（三）着力调整产业结构。加快培育现代产业体系，一、二、三产业增加值分别为72.1亿元、68.5亿元和109.4亿元，分别增长6.5%、15.3%和11%，三次产业比为29∶27∶44。　　　　加快发展特色现代农业。投入财政资金1531万元，推动“中国药都”建设。引进试种金银花、半夏、石斛等中药材1000多亩，建立种子种苗繁育基地7万亩。种植中药材116万亩，总产量24万吨。一方制药中间提取物、奇正藏药特色饮片及深加工等58个项目加快推进，完成投资33亿元。饮片加工能力达到20万吨，其中新增6万吨，实现加工产值30亿元。中医药产业总产值达到271亿元，增长69%。投入财政资金6420万元，扶持“中国薯都”建设。生产马铃薯脱毒原原种6亿粒，建立一、二级良种基地134万亩。种植马铃薯320万亩，总产量500万吨。薯香园公司马铃薯系列产品深加工、圣大方舟公司生物质基材创新创业示范园等项目进展较快，清吉淀粉公司年产3亿桶马铃薯无矾方便粉丝生产线建成投产，马铃薯加工能力达到60万吨。马铃薯产业总产值达到105亿元，增长34%。投入财政资金1.2亿元，扶持草食畜牧业加快发展。新发展规模养殖户1.4万户，引进投资千万元以上企业30家，畜禽饲养总量达到2500万头（只），增长38.9%，规模养殖比重提高到38%。草食畜牧业总产值达到80亿元，增长42.8%。投入财政资金3775万元，推动设施农业规模化发展。新建日光温室1070座、塑料大棚1.8万亩，蔬菜种植面积达到60万亩。积极推进农村区域发展综合示范区建设，创新农业经营模式，新增农民专业合作社1083个，规范提升100个，发展家庭农场12户，流转农村土地15万亩，累计达到70万亩。加强农机农艺融合、良种良法配套，推广以全膜双垄沟播为主的旱作农业389万亩。通渭县被国家农业部命名为全国产粮大县。粮食生产实现“六连增”，总产量达到153万吨。着力扩充第二产业。认真落实工业发展十大行动计划、倍增计划和非公有制经济发展措施，实施华能华家岭风电二期等千万元以上工业项目196个，陇西顺泰铝业年产10万吨铝型材、漳县祁连山水泥二期等65个项目建成投产，宏腾油气公司油气装备加工、甘肃招金难处理金精矿冶炼等131个项目完成年度计划，工业固定资产投资达到144亿元，增长37%。投入财政资金1.3亿元，扶持小微企业发展壮大，新增规模以上工业企业41户，累计达到122户，其中产值过亿元企业27户。完成工业增加值44亿元，其中规模以上工业增加值25亿元，增长19%，增速位居全省前列。投入财政资金6.3亿元，强力推进经济开发区和工业集中区建设，大力实施循环化改造，积极承接产业转移，新入驻企业53户，完成固定资产投资112亿元、增长35%，工业增加值29亿元、增长21.6%，实现税收3.7亿元、增长17%。新增建筑企业6户，入库企业累计达到73户，建筑业增加值25亿元，增长12%。致力推进第三产业转型发展。以建设华夏文明传承创新区为平台，论证储备重点项目127个，总投资594亿元。大力发展文化旅游业，积极参加第二届国际文化产业大会和第九届深圳文博会，成功举办中国·通渭国际书画艺术节、第二届中国·陇西李氏文化旅游节等大型节会，通渭书画、陇西李氏、岷县洮砚等文化产业园加快建设，完成投资15.8亿元，文化产业增加值4亿元、增长61%，旅游综合收入13亿元。定西马铃薯综合交易市场、陇西文峰药材交易城、首阳中药材产地交易市场跻身全省8大农产品批发市场之列，其中文峰药材交易城年交易额突破160亿元，成为全国第二大中药材专业批发市场和北方大宗中药材价格形成与信息发布中心。康美公司现代仓储物流及交易中心、西北金泽物流城等项目加快建设。农村金融综合改革开始破题，“12346”改革框架初步搭建。建设银行、甘肃银行在通渭和安定设立支行，农业银行建成惠民服务点1300多个，农村信用合作社建成金融服务便民点280多个，61%的行政村就近享受到了基本金融服务。发放农村小额信用贷款18.5亿元，增长89%。创新担保抵押方式，全力破解产业发展资金瓶颈，中药材仓单质押贷款取得突破，草食畜牧业保证保险贷款首创全省银保合作新模式。

　　（四）统筹加快城乡建设。坚持做大城镇，加快城乡一体化发展，城镇化率提高2.3个百分点，达到28.1%。定西市城市总体规划完成中期评估，市区、各县城控制性详细规划制定率分别达到95%和90%，乡镇总体规划实现全覆盖，村庄规划编制覆盖率提高到68.5%。实施各类城建项目230个，完成投资95.5亿元。同步推进市区新城建设与旧城改造，实施项目66个,完成投资45.5亿元。政府北路、小碱沟治理等18个基础设施项目基本建成，定西公园、中和教育港等25个项目快速推进，西岩新村、南环路两侧旧城改造等23个项目进展顺利。各县城及小城镇建设完成投资50亿元。城镇供水、供热及污水、垃圾处理等市政设施得到新改善，综合承载功能明显增强。建成农村公路通畅工程907公里，建制村通沥青或水泥路率达到58.8%。引洮供水一期定西市支渠及配套工程建设进入收尾阶段，田间配套工程全面建成，支渠、城乡供水水厂工程基本建成，累计完成投资21.9亿元。修编完成引洮一期农村供水工程调概报告，获批调概投资2.9亿元。实施中小河流治理等水利工程34项，完成投资7.2亿元，新增节水灌溉面积3万亩，解决了农村25万人的饮水安全问题。新修梯田28.3万亩，新增水土流失综合治理面积371.5平方公里。完成重点林业工程26.4万亩，其中天定高速公路等通道绿化2.8万亩。实施土地开发整理项目18.4万亩，新增耕地1.9万亩。

　　（五）全面发展社会事业。围绕提振教育教学质量，深入开展专题调查研究，召开全市教育工作陇西现场会议，制定出台办好人民满意教育意见和教师培训、基础教育基本建设、市区中小学布局等规划。高考录取率68%，其中二本上线率26.7%，提高3.5个百分点。财政投入教育经费29.6亿元，新建改扩建幼儿园、中小学、职业中学364所，消除D级危房25.5万平方米。加强教师队伍建设，引进紧缺专业教师120名。甘肃中医学院定西校区挂牌成立并开始承担药学类专业本科教学工作。与兰州中和投资创业集团共建职业技术学院进展良好。财政投入医疗卫生经费13.3亿元，市人民医院和陇西、渭源县中医院迁建工程基本完成，新建村卫生室133个。医药卫生体制改革深入推进，医疗机构全部配备使用基本药物并实行网上集中招标采购和零差率销售。陇西、临洮公立医院综合改革试点全面启动。中医药服务水平不断提高，陇西、岷县、临洮争创为全国农村中医药工作先进单位，通渭县争创为全省中医药工作先进县。新农合人均筹资标准由290元增加到340元，付费方式实现“一卡通”，参合率保持在95%以上。积极推进城乡居民大病医疗保险试点，赔付6722件、3117万元。争取香港“健康快车”第三次停靠我市，为1500多名贫困白内障患者免费实施了复明手术。紧贴产业开发深化科技体制改革，启动实施道地中药材产业化推广及惠民示范工程，定西市农业科学研究院成立运行。定西“百姓舞台”机制建设被列为全国公共文化服务体系建设示范项目，市县区广播电视农村覆盖网试点顺利启动。市级体育运动中心建设加快推进，全民健身活动广泛开展。全面落实计划生育优先优惠政策，低生育水平持续稳定。食品药品监管、工商行政管理、质量技术监督、公共资源交易等管理体制改革顺利完成，常态化市场监管机制基本建立。深入开展安全生产大检查和“打非治违”专项行动，不断靠实政府属地管理、部门日常监管和企业主体责任，安全生产形势稳定好转。健全完善应急管理体系，加强应急救援综合演练，在全省率先制定出台暴雨冰雹山洪地质灾害预警管理办法，应急救援和防灾减灾能力不断提高。《定西地区志》正式出版发行。国防动员和双拥工作得到加强。统计、物价、工商、质监、气象、民族宗教、外事侨务、国防后备力量建设等工作和妇女儿童、老年人、青少年、残疾人、社会慈善等事业都有了新的发展。

　　（六）不断增进民生福祉。全力保障和改善民生，财政用于民生方面的支出达到86亿元，增长52%。为民办理的10件实事全面完成。城镇新增就业2.7万人，其中高校毕业生7300多人。新增小额担保贷款4亿元，扶持创业8600多人，带动就业1.7万人。全力保障困难群众基本生活，城市、农村低保标准分别提高15%、28%，农村五保人均供养标准提高18%，发放临时救助和困难群众一次性生活补贴3亿元。重特大疾病救助实现城乡全覆盖，为3.1万城乡困难群众发放救助金7752万元，为57.4万城乡低保户和农村五保户代缴参合参保金1238万元。城镇职工各项社会保险稳健运行，城乡居民社会养老保险覆盖面不断扩大。为9724户城市住房困难家庭发放廉租住房租赁补贴1934万元，新开工建设保障性住房3289套，改造农村危房2.3万户。加强扶贫开发工作，实施易地扶贫搬迁1.6万人。扎实开展“双联”行动，发放“双联”贷款7.1亿元，社会各界投入帮扶资金20.5亿元，贫困村富民产业培育、生产生活条件改善取得新进展。培训城乡劳动力21.5万人次，输转68.7万人，劳务收入87.2亿元。农村贫困人口人均纯收入达到2382元、增长18%。减少贫困人口13.7万人，贫困面下降5个百分点。不断加强和创新社会治理，扎实推进平安定西建设，积极推行“两个全员参与”机制，高度重视群众来信来访，深入排查化解各类矛盾纠纷，全面构建社会治安防控体系，严厉打击各类刑事犯罪，社会大局保持和谐稳定。

　　（七）切实加强自身建设。全面落实党中央和省、市委关于改进工作作风密切联系群众的各项规定，深入推进“效能风暴”和“效能问责”行动，认真开展“政策规划落实年”和“管理服务创新年”活动，对工作落实不力、行政不作为慢作为乱作为的69个单位和143名工作人员进行了严肃问责。定期向市人大及其常委会报告工作，主动加强与市政协联系，95件市人大代表建议和142件市政协委员提案按期办结。修订完善市政府工作规则，进一步规范决策程序，提高依法行政效能。严格执行市政府常务会议会前学法制度，坚持不懈在政府常务会议上学习有关法律法规。修订规范性文件管理办法，全面落实规范性文件合法性审查和报送备案制度。取消、调整和下放市级行政审批项目101项。加强政府信息公开，完善农村信息公开平台，21个“数字农村”试点乡镇完成任务。电子民生平台办理群众诉求3.8万件次，解决了群众反映的一些实际问题。组建成立市公共资源交易中心，对政府集中采购、工程建设招投标、土地矿产及国有产权出让等一系列交易活动实行统一规范管理。加强行政监察和审计监督，严格执行《党政机关厉行节约反对浪费条例》，全面启动公务卡改革试点，大力推行预决算公开，“三公”经费支出实现了中央和省上规定的压减目标。

　　各位代表！过去的一年，我们遭遇的困难前所未有。接连不断的自然灾害，特别是“7.22”岷县漳县6.6级地震发生后，党中央、国务院和省委、省政府高度关切、鼎力支持，全市各级党政组织快速反应、紧急行动，广大干部以身作则、奋勇争先，部队官兵和公安民警舍生忘死、冲锋在前，社会各界慷慨解囊、捐款捐物，灾区群众不等不靠、奋力自救，各级新闻媒体积极呼吁、忘我工作，团结一心，众志成城，夺取了抗震救灾的重大胜利！

　　各位代表！过去的一年，我们面临的经济形势异常严峻。针对经济增速放缓，我们认真分析研判，采取切实措施稳定经济增长，有效缓解了经济下行压力。围绕重点工作任务，我们不断靠实责任，严格实行目标管理，做到前有目标计划、中有跟踪督导、后有考核评价，不间断、链条式推进工作。着眼破解发展难题，我们不断拓宽思路，顺应产业转移新趋势，创新就地举办产业节会招商，主动“走出去”上门推介招商，并建立跟踪服务和领导包抓推进机制，引进和落地建设了一批大项目、好项目。　　　　各位代表！过去的一年，全市经济社会发展取得的一切成绩，是市委正确决策、坚强领导的结果，是市人大、市政协主动监督、倾力支持的结果，是各级干部和广大群众团结奋斗、顽强拼搏的结果。在此，我代表市人民政府，向奋战在各条战线的广大干部群众，向各人民团体、民主党派、工商联和无党派人士，向人民解放军、武警官兵和中央、省驻定单位，向所有关心支持定西发展进步的各界人士，表示衷心的感谢和崇高的敬意！

　　当前，定西正处在扶贫攻坚、后发赶超、转型跨越的关键时期。我们清醒地认识到，全市经济社会发展还存在不少困难和问题，主要是：防灾减灾和灾后恢复重建任务艰巨。农村贫困程度深、贫困面大，扶贫攻坚任务非常繁重。城乡基础设施建设欠账大，建设用地整体比较紧张。支撑长远发展、带动经济结构调整的大项目和生产性项目还比较少。产业结构不合理，一产占比大，二产总量小，三产整体实力不强。项目建设特别是中小企业融资难问题依然突出。城乡劳动力创业就业技能缺乏，持续增收难度较大。教育、卫生、文化等公共服务设施建设比较滞后，服务水平不高。社会治安、安全生产形势不容乐观。发展体制机制不活，少数部门和干部创新意识、服务意识、责任意识不强，发展环境有待不断优化。对此，我们要高度重视，扎实努力，采取更加有效的措施，认真加以解决。

　　2014年主要任务

　　今年是全面深化改革的第一年，也是加快实施“十二五”规划的关键一年。政府工作的总体要求是：全面贯彻落实党的十八大、十八届三中全会精神，坚持以科学发展观为指导，以推进扶贫开发为核心，以转变经济发展方式为主线，以改革创新为动力，紧紧围绕打造“中国药都”、“中国薯都”和全省草食畜牧业大市，坚持不懈促进产业转型升级、工业跨越发展、城镇做大做强，坚定不移调整优化结构、扩大经济总量、提升发展水平，全面加快农业现代化、工业化、城镇化和信息化进程，努力与全省全国同步进入全面小康社会。　　　　经济社会发展的主要预期目标是：生产总值增长11%左右，固定资产投资增长30%，规模以上工业增加值增长16%，社会消费品零售总额增长15%，大口径财政收入增长18%，公共财政预算收入增长15%，城镇居民人均可支配收入增长15%，农民人均纯收入增长17%。居民消费价格涨幅控制在3.5%以内，人口自然增长率控制在7‰以下，城镇登记失业率控制在4%以下，节能减排控制在省下达指标之内。

　　围绕上述要求和目标，重点抓好以下工作：

　　（一）加快灾后重建进度，建设幸福美好家园。严格按照岷县漳县地震灾后恢复重建总体要求，统筹长远发展与近期建设，重点抓好7个小城镇、48个集中安置点、15个重灾村和192个国省道沿线村庄风貌改造等规划设计，加紧编制岷县梅川镇、茶埠镇及县城，寺沟乡及火车站，漳县大草滩火车站小城镇和遮阳山景区，三岔镇、殪虎桥乡一体化发展规划，2月底前完成项目规划设计并与群众讨论形成一致意见，3月底前完成各项开工准备工作，4月份开工建设，8月底前完成受灾小学、幼儿园恢复重建，年底前完成受灾中学、乡村卫生院（室）等公共服务设施和城乡居民住房重建。启动实施国道316线陇西至会川段、省道306线岷县茶埠至马坞段、省道210线岷县巴仁口至铁尺梁段等交通基础设施重建项目。全面完成“5.10”岷县特大冰雹山洪泥石流灾后恢复重建任务。切实加强质量安全和资金使用全程监管，确保高效廉洁重建。

　　（二）扎实推进项目建设，着力扩大投资规模。深入实施“3341”项目工程，坚持扩充总量与优化结构并重，紧盯国家政策及产业投资导向，筹措前期经费6000多万元，围绕基础设施改善、扶贫攻坚、结构调整以及丝绸之路经济带建设、特色优势产业开发和现代服务业发展，谋划千万元以上项目1200多个，进一步充实项目储备库。坚持“走出去”与“引进来”相结合，深入开展“千亿元大招商”活动，优化招商落地服务，市直部门完成招商引资任务300亿元，各县区分别完成100亿元。争取招商引资到位资金400亿元以上，增长35%。加大项目争取力度，落实国家政策性资金70亿元以上。抓紧实施《甘肃定西渭河源区生态保护与综合治理规划》，力争《定西市生态文明建设规划》在国家层面获批。实施500万元以上项目1700个，力争完成投资660亿元以上。配合抓好兰渝铁路、宝兰客专、临渭和渭武高速公路等省部属重大项目建设，力争国道212线临洮水泉至漳县殪虎桥段、通渭至榜罗二级公路开工建设。按照全省项目观摩“看高不看低”要求，集中力量组织实施一批高新技术项目。协调抓好甘肃东化新材料、定西西部汽车城、香港彬立汽车产业园等重点项目建设。

　　（三）创新扶贫开发模式，加快脱贫致富步伐。以扶贫工作重点乡镇、贫困村为主战场，深入实施“1236”扶贫攻坚行动计划，努力在持续增加收入和解决不愁吃、不愁穿方面，探索建立产业、技能和城镇化带动机制，夯实扶贫攻坚的增收基础；在强化义务教育、基本医疗和住房保障，以及实现基础设施建设、富民产业培育、易地扶贫搬迁、金融资金支撑、公共服务保障、能力素质提升突破等方面，探索建立多渠道增加投入机制，增强扶贫攻坚的基础保障能力。始终坚持以市场为导向，充分发挥区域性资源比较优势，大力培育和发展“一乡一业”、“一村一品”富民产业。加强财政扶持资金使用监管，建立以奖代补激励促进机制，鼓励和引导享受财政扶持的各类龙头企业增强社会责任，积极到贫困乡村建立种养基地，吸纳贫困农民务工就业，形成利益共同体。整合各类培训资金，创新教育培训方式，以职业技术学校为主，培训“两后生”5000多人，输转剩余劳动力65万人，实现劳务收入80亿元以上。加大财政转移支付争取力度，增列扶贫专项预算，抓好“中和农信”小额信贷和世界银行第六期贷款项目，全面落实国家扶贫贴息贷款政策，加强贫困村互助资金运行监管，千方百计拓宽扶贫投入渠道。充分利用国开行贷款，加快推进以农村公路、安全饮水为重点的各项基础设施建设，认真抓好通渭扶贫攻坚交通先行试点县工作。以促进动力用电率先覆盖贫困乡村为目标，加快实施农村电网改造工程。优化贫困乡村发展规划，对发展潜力较大的乡实施撤乡建镇，结合产业培育开发、易地扶贫搬迁、市场改造建设和交通道路完善，规划建设小城镇，形成带动扶贫开发的区域经济中心。强化“双联”单位扶贫责任，充分发挥行业扶贫和社会帮扶作用，努力构筑“大扶贫”工作格局。完成整村推进项目60个以上，易地扶贫搬迁1.4万户7万人，确保10个扶贫工作重点乡镇、138个贫困村脱贫，减少贫困人口17.3万人。

　　（四）大力发展现代农业，提升特色优势产业开发水平。按照依法自愿有偿原则，鼓励和引导农村土地向种养大户、家庭农场、专业合作组织、农业产业化龙头企业流转。立足特色优势产业开发，大力培育发展家庭经营、集体经营、合作经营和企业经营，以新型农业经营主体助推现代农业建设。围绕“中国药都”建设，建立种子种苗繁育基地5万亩、标准化药源基地60万亩，示范扩大金银花等中药材种植面积，力争岷归等道地药材创建为中国驰名商标，陇西县创建为全国中药材产业知名品牌示范区。加快陇西中医药循环经济产业园循环化改造。陇西、岷县、渭源等县以县城投公司为主体，建设农户加工中药饮片园区，并以成本价为农户办理产权证和土地使用证，努力把家庭作坊式加工转变为工厂化、标准化、规范化生产。新增中药饮片GMP认证企业5户、GSP流通企业20多户，新建中药提取物生产线6条。饮片加工能力扩大到30万吨。积极开发中医保健、日化和制药产品，不断提高附加值。力争中医药产业总产值达到380亿元。围绕“中国薯都”建设，突出良种繁育和精深加工“两头建设”，组建马铃薯种薯产业集团，脱毒原原种生产能力达到7亿粒，一、二级良种基地达到140万亩，形成良种梯级繁育体系。以多用途工业变性淀粉产品、高档休闲食品开发和循环利用为主攻方向，鼓励扶持加工企业加强技术改造，延长产业链，力争安定区争创为全国马铃薯产业知名品牌示范区。马铃薯产业总产值达到120亿元。结合“两都”建设，积极推进地方名优产品商标注册工作，提高当地商品市场占有率和附加值。把草食畜牧业作为扶贫攻坚的战略性产业，加快全省草食畜牧业大市建设。多渠道投入资金30亿元，扩大玉米秸秆青贮规模，改进技术服务，完善市场流通，新扶持发展规模养殖户2万户、家庭养殖场2000个、规模养殖场400个、养殖专业合作社100个，培育养殖专业村120个，争创省部级标准化养殖示范场6个。积极引进培育加工龙头企业，扶持现有龙头企业发展壮大。草食畜牧业总产值达到120亿元。充分利用引洮水资源，加快发展绿色无公害蔬菜和小杂粮、林果等区域性特色产业，新增以日光温室、塑料大棚为主的设施农业1.9万亩，以高原夏菜为主的蔬菜种植面积达到65万亩。高度重视粮食生产，粮食总产量稳定在140万吨左右。加强粮食储备，确保全市粮食安全。加快建设农村区域发展综合示范区，不断扩大农业机械覆盖面，完成全膜双垄沟播种植384万亩。

　　（五）推动工业持续健康发展，加快产业升级步伐。着力打造千亿级产业、百亿级园区、十亿级企业，深入推进工业发展十大行动计划和倍增计划，实施千万元以上工业项目136个，重点抓好甘肃招金难处理金精矿冶炼、华家岭风电三期等4个投资10亿元以上和中盛铝业25万吨铝型材、江能医药公司中药材综合加工等40个投资亿元以上项目建设，力争陇西一方科技产业园、蓝天年产20万吨淀粉糖等45个项目建成投产，普尔康道地药材精深加工等91个项目完成年度计划，工业固定资产投资增长25%。扶持小微企业提升产能、扩大规模、增加效益，新增规模以上工业企业25户，其中产值过亿元企业10户以上。投入财政资金2.6亿元，加快全市经济开发区和工业集中区循环化改造，加大扩容升级力度，统筹“一区多园”建设，新引进入驻企业50多户，积极申报争取定西经济开发区升格国家级开发区。严格企业入园标准，促进土地节约集约高效利用。鼓励工业企业互保共建市场，积极发挥技术创新主体作用，改进生产工艺，研发新产品，推动中医药和马铃薯精深加工、铝冶炼及深加工、矿产建材及新能源开发、装备制造等主导产业转型升级，带动经济结构调整。全面推进质量振兴工作，加强品牌建设，鼓励企业争创省、市政府质量奖。

　　（六）壮大提升第三产业，增强经济发展活力。抢抓国家支持现代服务业发展机遇，力促第三产业提质增效。紧贴丝绸之路经济带建设，充分发挥交通区位优势，大力发展现代物流，加快定西丝绸之路国际商贸物流城、中邦华运物流园、金帆现代城市流通综合体等项目前期工作，加快定西荣芳汽配城、西北金泽物流园等商贸物流项目建设进度。积极培育工业研发设计、电子商务、工程咨询等新型业态，促进生产性服务业更快发展。实施“十园百市千店”工程，新建或改扩建农贸市场10个、乡镇商贸中心10个、村级商品服务社380个。推进文化旅游产业深度融合发展，加快临洮马家窑彩陶、通渭书画、陇西李氏、岷县洮砚等文化产业园及临洮卧龙湾休闲水镇等重点文化产业项目建设，启动实施临洮太石水城一期工程，文化产业增加值增长30%。抓好贵清山、渭河源等景区基础设施建设，积极开发富有地域文化特色的旅游产品，旅游综合收入增长25%。用足用活省市促进非公有制经济发展政策，促进个体私营经济转型升级。完善农村金融服务体系，推动金融机构在县区合理布局，加快乡村金融网点建设。积极吸引银行、证券、保险和股权投资机构到定西设立分支机构。加快“数字农村”建设，拓展服务功能，推进农村信息公开平台与金融服务有效融合，促进农村信用贷款稳步增长。围绕特色优势产业发展、农村经济改革重点破解担保难题，加大农民和小微企业融资支持力度。推进中药材种植保险等特色农业保险试点，探索开展农房保险等涉农保险。加强银证保合作，探索建立银行信贷、直接融资与保险保障融合机制。认真落实支持服务业发展各项政策，服务业增加值增长11%。

　　（七）强化基础设施建设，积极推进新型城镇化。深入贯彻党中央、国务院和省委、省政府关于加快新型城镇化的战略部署，坚持高起点规划、高水平建设、高效能管理，突出布局优化、功能配套和产业支撑，统筹推进城镇道路、供热供水、供电供气、垃圾污水处理和湿地水景、绿化景观等基础设施建设，完善教育、卫生、文化、体育等公共服务体系和工业发展、市场物流等产业功能区，着力增强综合承载发展功能，逐步形成市区、县城和建制镇优势互补、合理布局、连接畅通、特色鲜明的发展新格局。继续完善市区和各县城规划编制，适时启动定西市城市总体规划修编和市区市场建设、商业网点、城镇特色风貌规划编制，争取村庄规划覆盖率提高20个百分点。定西市区实施项目86个，完成投资50亿元。新城区重点启动建设湿地公园、电网工程、亚行贷款城市道路和关川河景观带改造等项目，加快污水处理、供水管网、体育馆等公用设施建设进度,完成市人民医院、市行政中心等单位搬迁，确保定西展览馆和文化艺术中心等项目按期投入使用。旧城区围绕完善功能，重点抓好南山根安居小区集中供热工程、定西宾馆周边等片区和永定路市场、东关市场、再就业市场等改造项目，加快实施南环路、教育东路等项目建设，全力打通断头路，构建新旧城区顺畅通达的路网体系。各县城和重点小城镇实施项目191个，完成投资60亿元。创新城镇建设投融资机制，降低城镇建设准入门槛，推进城镇建设投资主体多元化和市政公用设施运营市场化。深化户籍制度改革，全面放开县城和建制镇落户限制，加快农业转移人口市民化，力争城镇化率提高到30%以上。坚持建管并重，切实加强城市管理，建立长效机制，认真解决冬季供暖、物业管理、交通秩序、市容市貌、临街经营等方面存在的突出问题。深入开展精神文明创建活动，不断提高市民素质和城市文明程度。全力抓好引洮供水一期支渠及配套工程扫尾建设和质量监控，确保4月底前完成管道打压试水，6月份与主体工程同步建成通水。抓紧制定引洮支渠及配套工程运营管理办法，确保工程全面稳定发挥效益。建设河道治理等重点水利工程27项，农村安全饮水入户率达到70%以上。实施甘肃东部百万亩土地整治重大工程定西片区项目，新增耕地1.9万亩。

　　（八）全面深化各项改革，推进体制机制创新。认真贯彻党中央和省、市委关于全面深化改革的重大部署，着力推进重点领域和关键环节改革，破除制约发展的体制机制障碍。深化行政审批、投融资体制和工业领域改革，减少政府对微观经济活动干预，凡是市场机制能够有效调节的经济活动一律取消审批，企业投资项目除国家明文规定外，一律由企业依法依规自主决策。积极推进政府机构改革，探索建立事业单位法人治理结构，全面完成市、县区事业单位分类改革，严控机构编制，确保人员编制零增长。加快推进财政管理体制改革，扩大绩效预算试点，实施全面规范、公开透明的预算制度。合理划分市与县区的事权与支出责任。建立规范合理的政府债务管理及风险预警机制。坚持和完善最严格的耕地保护制度，在符合规划和用途管制前提下，允许农村集体经营性建设用地出让、租赁、入股，实行与国有土地同等入市、同权同价。加快农民住宅产权确权登记，保障农民宅基地用益物权。制定农村空置宅基地退出补偿办法，拓宽农民增加财产性收入渠道。规范征地程序，健全被征地农民合理、规范、多元保障机制。完善土地租赁、转让、抵押二级市场。全面推进水利改革试点，深化集体林权配套制度改革。完善以县为主的教师管理体制，探索建立教育管理激励、约束、保障机制和校长、教师引进交流机制。继续深化医药卫生体制改革，稳步推进公立医院综合改革，完善基层医疗卫生单位收入分配机制。探索市、县区医院同省内外大医院、医学院合作交流的方式和途径，加快县乡医院一体化改革。完善覆盖城乡的中医医疗服务体系。坚持计划生育基本国策，落实一方是独生子女的夫妇可生育两个孩子政策。建立最严格的覆盖全程的食品药品监管制度，落实食品原产地可追溯制度和质量标识制度，保障食品药品安全。探索中小微企业专利权质押融资模式，建立重大科技项目市县区与企业共投共享机制，加快科技创新平台建设，组织实施国家科技惠民计划，引导更多企业成为技术创新主体。

　　（九）加强生态文明建设，持续改善人居环境。抢抓省上建设国家生态安全屏障综合试验区的有利机遇，全面加强生态建设和环境保护，努力打造黄土高原水土保持示范区和全国生态文明示范区。加快构建循环型产业体系，抓好通渭国家级循环经济示范县建设，争取安定区列入全省循环经济示范县（区）。新修梯田30万亩，综合治理水土流失面积350平方公里。以城镇面山、交通干道沿线、引洮灌区及村庄集镇为重点，整区域推进造林绿化。结合 “百镇千村”示范工程实施，完成15个重点镇、145个示范村的绿化建设。抢抓国家恢复退耕还林政策机遇，扩大退耕还林规模。新增造林封育24万亩，森林覆盖率达到14.9%。加强饮用水源地保护，完成各级水源地评估整改工作。加大重点领域污染治理力度，加快城镇污水处理工程及配套管网建设，强化企业污染深度治理，严格新上项目环评审批。落实养殖污染减排措施，确保30%以上畜禽规模养殖场和养殖小区达到国家减排标准。大力推广标准化农膜生产应用，强化废旧地膜、烂尾菜、生活垃圾处理利用，废旧农膜回收利用率达到70%。建立完善环境监测预警体系，强化节能减排目标考核。实施重点节能改造项目，淘汰落后产能。加大城乡环境和交通秩序整治力度，持续改善人居环境，促进美丽宜居乡村建设。

　　（十）全力发展社会事业，切实保障和改善民生。全面落实市委、市政府关于办好人民满意教育的意见，推进义务教育学校标准化建设，着力改善薄弱学校基本办学条件。加强教育教学质量管理，教师培训比例提高16个百分点，引进紧缺专业教师100名，确保高考应届生二本上线率提高3个百分点。创新职业教育办学模式，申报设立高等职业技术学院，强化校企校地合作办学，中等职业教育就业率达到95%。加大协调力度，推动甘肃中医学院定西校区建设取得实质性进展，西北师大附属中和学校秋季实现高中、小学、幼儿园招生。引导组建农民文化专业合作社，建设村级文化活动室200多个。争取实施乡镇卫生院职工周转宿舍和全科医生培养基地项目，新建村卫生室240多个。实施中医药服务能力提升工程，争取安定区创建为国家或省级中医药工作先进单位。面向农村大力培养全科医生，努力提高基层医疗卫生服务能力。加强食品药品监管队伍能力建设，积极探索乡镇食品药品有效监管方式。提高城乡居民最低生活保障水平，健全完善社会救助制度。加大农民工工资清欠力度，全面落实农民工工资保证金制度。积极发展体育事业，提高竞技水平，推进全民健身。全面靠实各级责任，切实强化全方位监管，促进安全生产形势持续稳定好转。加强电子民生平台建设，探索有效解决群众合理诉求机制。创新社会治理方式，激发社会组织活力，重视和加强信访工作，完善社会矛盾纠纷预防化解机制。加快打造数字化、高清化、智能化、网格化视频监控系统，推进公共安全体系建设。保障妇女儿童合法权益，健全残疾人就业服务和社会保障体系。加强双拥共建、国防后备力量建设及外事侨务、民族宗教、防震减灾、气象服务、公共节能等工作，推动老年人、青少年、社会慈善等事业健康发展。

　　各位代表！今年市政府将筹措资金8.5亿元，继续为群众办好10件实事：1.改造建设校舍3.8万平方米，改善63所农村薄弱学校办学条件。2.改扩建乡镇卫生院13所；为137所乡镇卫生院培训急诊急救医师150名；免费为2.5万名新生儿进行遗传性疾病筛查。3.改造农村危房1.8万户；新增农村沼气用户1740户。4.建成农村公路通畅工程960公里，行政村通水泥或沥青路率达到65%以上。5.为困难群众办理法律援助案件800件。6.新增小额担保贷款3亿元，支持6000人自主创业；培训城乡劳动力15万多人次。7.建成安定区李家堡和陇西县机动车辆检测站，方便群众办理车辆检测业务。8.将100个贫困行政村列入互助资金试点，进行重点扶持。9.解决农村10万人安全饮水问题。10.治理市区旧城区背街小巷10条2.2公里；改建公交站点42个；在公园路中小学、东方红中学路口修建过街天桥2座，对新旧城区市政设施、绿化景观、环境卫生进行综合整治。

　　加快建设法治政府和服务型政府

　　全面完成今年改革发展稳定各项工作任务，推动经济社会持续健康较快发展，政府肩负的责任重大而艰巨。我们将按照党中央、国务院和省委、省政府及市委关于全面深化改革的部署要求，结合深入开展党的群众路线教育实践活动，加快转变职能，改进工作作风，优化组织结构，加强自身建设，全面正确履行职责，努力建设法治政府和服务型政府。

　　（一）加强理论学习。健全完善理论学习制度，深入学习党的十八大、十八届三中全会和习近平总书记系列重要讲话精神，学习法律法规、各项政策和市场经济、公共管理等知识，不断提高政府领导和工作部门负责人用理论指导实践的能力，用法律政策推动工作、管理社会事务的能力。紧密结合政府工作实际，建立理论务虚制度，深入分析形势，认真研究事关发展的重大问题，不断提升政府领导和工作部门负责人廓清工作思路、破解发展难题和狠抓任务落实的能力。建立完善政府工作人员教育培训机制，有针对性地开展能力素质培训，不断提升政府工作人员服务发展的业务素质和综合能力。

　　（二）加强依法行政。自觉接受人大依法监督和政协民主监督，认真执行人大决议、决定，及时落实人大审议意见，高度重视并自觉接受舆论监督和社会公众监督。健全科学民主决策机制，对重大决策开展咨询、听证、公示、风险评估，建立规范性文件和政府签订合同法律审查制度。加强食品药品、市场监管、安全生产、环境保护、劳动保障、农资供应等领域执法力量和执法能力建设。严格实行行政执法责任制和执法过错责任追究制，坚决禁止变相设定行政许可，依法治理乱收费、乱罚款、乱检查行为，为经济社会发展创造良好法治环境。

　　（三）加强作风建设。认真贯彻落实党中央和省、市委关于改进工作作风密切联系群众的各项规定，从着力改进会风文风、切实精简文件简报、严格公务接待和规范评比检查入手，坚决反对形式主义、官僚主义、享乐主义和奢靡之风，把更多时间和精力集中到研究发展问题、推动工作落实上来。扎实开展党的群众路线教育实践活动和“双联”行动、“效能风暴”行动，强化纪律作风整治，重点整治一些部门和工作人员纪律散漫、作风漂浮、吃拿卡要、推诿扯皮等突出问题，建立高效办事、扎实工作的常态化机制。　　　　（四）加强廉政建设。认真贯彻落实党中央和省、市委关于推进惩治和预防腐败体系建设的各项部署，加强廉政宣传教育，主动接受各方面监督，筑牢拒腐防变的思想道德防线。健全风险预警和权力运行监控机制，强化对工程建设、土地管理、产权交易、政府采购等重点领域行政监察和审计监督，切实把权力关进制度的笼子里。严格执行《党政机关厉行节约反对浪费条例》，倡导艰苦奋斗、勤俭节约的良好风尚，坚决反对铺张浪费。巩固清理办公用房、清退超标车辆、停建楼堂馆所等专项整治成果，继续大力压减“三公”经费支出，营造节俭办事、廉洁从政的清新政风。

　　各位代表！回顾过去，我们不畏艰险、奋力拼搏；瞻望未来，我们信心百倍、勇往直前。让我们更加紧密地团结在以习近平同志为核心的党中央周围，在省委、省政府和市委的坚强领导下，在市人大、市政协的监督支持下，改革创新、求真务实，开拓进取、扎实工作，奋力完成各项目标任务，为打好扶贫攻坚战、推动经济社会持续健康发展、建设幸福美好新定西作出新的更大的贡献！
